# Impact of behavioural risk factors on death within 10 years for women and men in their 70s: absolute risk charts

**DOI:** 10.1186/1471-2458-12-669

**Published:** 2012-08-17

**Authors:** Annette Dobson, Deirdre McLaughlin, Osvaldo Almeida, Wendy Brown, Julie Byles, Leon Flicker, Janni Leung, Derrick Lopez, Kieran McCaul, Graeme J Hankey

**Affiliations:** 1 The University of Queensland, School of Population Health, Herston, Australia; 2 Western Australian Centre for Health & Ageing, CMR, Western Australian Institute for Medical Research, Perth, Australia; 3 School of Psychiatry & Clinical Neurosciences, University of Western Australia, Perth, Australia; 4 Department of Psychiatry, Royal Perth Hospital, Perth, Australia; 5 The University of Queensland, School of Human Movement Studies, St Lucia, Australia; 6 The University of Newcastle, Centre for Gender, Health and Ageing, Newcastle, Australia; 7 School of Medicine and Pharmacology, University of Western Australia, Perth, Australia; 8 Stroke Unit, Department of Neurology, Royal Perth Hospital, Perth, Australia; 9 The University of Queensland, School of Population Health, Herston Road, Herston, Queensland 4006, Australia

**Keywords:** Mortality, Risk factors, Men, Women

## Abstract

**Background:**

Estimates of the absolute risk of death based on the combined effects of sex, age and health behaviours are scarce for elderly people. The aim of this paper is to calculate population based estimates and display them using simple charts that may be useful communication tools for public health authorities, health care providers and policy makers.

**Methods:**

Data were drawn from two concurrent prospective observational cohort studies of community-based older Australian women (N = 7,438) and men (N = 6,053) aged 71 to 79. The outcome measure was death within ten years. The predictor variables were: sex, age, smoking status, alcohol consumption, body mass index and physical activity.

**Results:**

Patterns of risks were similar in men and women but absolute risk of death was between 9 percentage points higher in men (17 %) than in women (8 %) in the lowest risk group (aged 71–73 years, never smoked, overweight, physically active and consumed alcohol weekly) and 21 % higher in men (73-74 %) than women (51-52 %) in the highest risk group (aged 77–79 years, normal weight or obese, current smoker, physically inactive and drink alcohol less than weekly).

**Conclusions:**

These absolute risk charts provide a tool for understanding the combined effects of behavioural risk factors for death among older people.

## Background

The demographic changes and increasing life expectancy in developed countries such as Australia mean that increasing numbers of people will live into their 70s. Simple, valid information about risk of death based on sex, age and health behaviours may be useful for public health authorities, health care providers and policy makers.

Studies that have estimated the long-term prognosis for mortality among the elderly show that modifiable behavioural risk factors such as smoking, body mass index (BMI), alcohol consumption, and physical activity are major determinants [1-9]. However many of these studies have only examined these risk factors individually and often in one sex only or in people with a wide ranges of ages.

While each of these risk factors has an effect on mortality, the combined effect of these risk factors is cumulative. It has been estimated that for each 2-point increase in mean BMI and each 10 % increase in smoking prevalence among overweight adult men, there is reduction in life expectancy of around one year [10]. Among women aged 34–59, smoking, overweight BMI, and limited physical activity each predicted mortality, and in combination resulted in a four-fold increase in all-cause mortality [11]. A study of the effects of individual and combined lifestyle risks on 20-year mortality among adults in the UK found that mortality doubled with one risk factor, and increased by three with two risk factors [12]. However, few studies have included significant numbers of older participants, nor followed them long enough to determine the combined effects of these behaviours at older ages.

For this paper the first aim was to provide estimates of the combined effects of several demographic and behavioural risk factors on all-cause mortality using valid observational data from two large Australian cohort studies of women and men in their seventies. The second aim was to display them using simple charts analogous to those commonly used in clinical practice for predicting cardiovascular disease [13-15].

## Methods

### Setting and participants

Data were derived from the Men, Women and Ageing project, which incorporates data from the 1921–1926 birth cohort of the Australian Longitudinal Study on Women’s Health (ALSWH) and from the Health in Men Study (HIMS). Detailed methods for both studies have been described elsewhere [16,17].

Briefly, the ALSWH is a repeated survey of the health and well-being of three cohorts of women who were born in 1973–1978, 1946–1951 and 1921–1926 when recruited in 1996. The women were randomly selected from the Australian national health insurance database (Medicare), which includes all citizens and permanent residents, with intentional over-sampling of women from rural and remote areas^16^. The project uses mailed questionnaires to collect self-reported data on health and related variables every three years. In the 1921–26 birth cohort, 39,000 women were initially invited to participate; of these 1,100 were not contactable and 2,366 were ineligible. Of the 35,534 remaining women, 12,432 responded to the first survey in 1996. Because the physical activity questions in the first survey differed from those in the HIMS survey, data used in the current analyses were drawn from the second survey conducted in 1999, to which 10,430 women responded. The ethics committees of the University of Newcastle and the University of Queensland approved the research protocol.

The HIMS cohort was formed from men screened for abdominal aortic aneurysm in a randomised controlled trial conducted in Perth, Western Australia in 1996. In this trial, eligible men were aged 65–83 years, resident in Perth (the capital of Western Australia), and not in long stay institutional accommodation. A list of eligible men was drawn from an electronic copy of the electoral roll in 1996 (enrolment to vote is compulsory for adult Australians) and, after excluding 8,801 who were no longer resident in Perth and 2,296 who had died before the study began, the remaining men were randomised into a screening group (n = 19,352) or a control group (n = 19,352). Of those invited to be screened, 1,836 were ineligible, 5,303 did not respond or refused, and 12,203 were screened between 1996 and 1999^17^. These 12,203 screened men formed the HIMS cohort and have been followed since their recruitment. The HIMS research protocol was approved by the ethics committee of the University of Western Australia.

### Measurements

A postal questionnaire was used to collect data from the women, and all variables, including height and weight and smoking status, were self-reported. Data from the men were obtained using a postal questionnaire that was reviewed during a face-to-face interview by a research nurse at a clinic visit. During this visit, physical measures including height and weight were taken, and participants were asked about their current smoking status.

Age was calculated from the date at which the survey forms were returned. Because the age range was much greater for the men (65 to 83 years) than for the women (71 to 79 years), those men outside the age range of the women were omitted from the analysis (N = 6068 omitted).

Smoking status was categorized as ‘never smoked’, ‘ex-smoker’ or ‘current smoker’. BMI was calculated from height and weight (kg/m^2^) and categorized according to WHO recommendations as ‘underweight’ (<18.5), ‘normal’ (18.5 to <25), ‘overweight’ (25 to <30) and ‘obese’ (> = 30) [18]. Due to small numbers of participants in the underweight group and the possibility that these people were already suffering from significant disease, they were excluded from the analysis (N = 87 men and N = 319 women).

Participants in both ALSWH and HIMS were asked about their usual frequency and quantity of alcohol consumed and very few of these older people reported drinking large quantities of alcohol. Comparable categories of alcohol consumption were derived for women and men [19]. These were: drinking alcohol less than weekly (including those who never or rarely drink), or drinking weekly or more often.

In both cohorts, participants were asked to report the duration of time spent in *vigorous leisure activity/exercise (that makes you breathe harder or puff and pant)* in the last week (women) or a usual week (men)*.* A MET (metabolic equivalent) value of 6 was applied to responses to these questions, in line with estimates for 'hard' physical activity in this age group [20]. In addition, the women were asked to report time spent *walking briskly (for recreation or exercise, or to get from place to place),* and in *moderate leisure activity (like golf, bowls, social tennis, moderate exercise classes),* while the men were asked to report on *non-vigorous exercise for recreation or health and fitness (e.g. slow walking, slow cycling, Tai Chi, yoga, etc.)* Responses to the women’s walking and moderate activity questions were combined to calculate time in non-vigorous exercise and a MET value of 3 was applied to the responses for non-vigorous activity from both men and women. A physical activity score was calculated from total minutes per week in each of the two categories of physical activity and the MET values: (non-vigorous minutes per week x 3.0 MET + vigorous minutes per week x 6.0 MET). Scores were categorized as ‘inactive’ (<600) or ‘active’ (≥600). The 600 MET.minutes/week threshold equates to 200 minutes of moderate activity/week, which is commensurate with current physical activity guidelines [21].

Due to the differences in data collection methods missing data were more common from the women than the men. All participants with missing data were excluded from the analyses. The missing data were as follows: for smoking, women n = 781, men n = 0; for BMI, women n = 1389, men n = 9; for alcohol, women n = 1294, men n = 482; and for physical activity, women n = 1045, men n = 25; with some people having missing data for more than one of these variables. The resulting analysis data set was for N = 7438 women and N = 6053 men.

### Outcome variable

The outcome variable was death, from any cause, within 10 years of collection of the risk factor data. For ALSWH participants, deaths were identified by matching identification information to the Australian National Death Index [22]. For HIMS participants, all of whom lived in Western Australia at recruitment, death information was obtained through the Western Australian Data Linkage System [23], which provides electronic linkage to the state’s population health data collections and includes records from the death register.

### Statistical methods

Logistic regression models were fitted for each sex separately. Each of the explanatory variables was treated as categorical and age (three categories), smoking status (three categories), BMI (three categories) and physical activity (two categories) were modelled simultaneously. The models were assessed using various measures of goodness of fit and were then used to estimate the predicted proportion of deaths within 10 years for each combination of explanatory variables. Absolute risk estimates were calculated by inverting logits of predicted proportions of deaths. Sensitivity analyses were conducted for the physical activity variable using different multipliers for the MET values for women and men separately and together. All analyses were performed using Stata/SE 11.0.

## Results

Characteristics of the women (N = 7438) and men (N = 6053) included in the analysis are shown in Table 1. There were 1734 deaths among the women and 2290 deaths among the men during the ten-year follow-up period.

**Table 1:** Characteristics of participants in the Australian Longitudinal Study on Women’s Health and the Health in Men Study cohorts

**Variable**	**Women (n=7438)**		**Men (n=6053)**		**p-value from chi-squared test**
	**N**	**%**	**N**	**%**	
Age (years)					<0.001
71-73	811	(10.9)	2605	(43.0)	
74-76	4688	(63.0)	2040	(33.7)	
77-79	1939	(26.1)	1408	(23.3)	
Smoking status					<0.001
Never smoked	4752	(63.9)	1621	(26.8)	
Ex-smoker	2328	(31.3)	3882	(64.1)	
Current smoker	358	(4.8)	550	(9.1)	
Body mass index (kg/m^2^)					<0.001
Normal (18.5 to <25)	3792	(51.0)	1939	(32.0)	
Overweight (25 to <30)	2593	(34.9)	3079	(50.9)	
Obese (30 or more)	1053	(14.2)	1035	(17.1)	
Alcohol consumption					<0.001
Weekly or more	2414	(32.4)	3812	(63.0)	
Less than weekly	5024	(67.6)	2241	(37.0)	
Physical activity (MET.mins/week)					<0.001
Inactive (<600)	4806	(64.6)	2725	(45.0)	
Active (600 or more)	2632	(35.4)	3328	(55.0)	

### Risk factors for death

Results of the logistic regression models are shown in Table 2. Both models fitted reasonably well: Hosmer-Lemeshow statistics for 10 groups, chi-square (8) = 11.97, p = 0.15 for women, chi-square (8) = 11.03 and p = 0.20 for men. The odds ratios for death within 10 years were similar for women and men for all the risk factors. But the probability of death was higher for men as indicated by the absolute risk estimates for women and men with the reference levels of the risk factors (calculated from the constant terms in the models). The risk difference (calculated from the difference in the constant terms) was 33 % (95 % confidence interval (CI): 27 % to 40 %). The odds of death increased substantially with age, and were more than twice as high for current smokers as for never smokers. For BMI, estimates were lower for the overweight than normal weight groups. The odds of death were higher for those who drank alcohol less than weekly and lower for those who were physically active. The results changed very little if different weights were used for the MET values used to calculate the physical activity variable.

**Table 2:** Results of logistic regression models for probability of death within 10-years for participants in the Australian Longitudinal Study on Women’s Health and the Health in Men Study cohorts: odds ratios and 95 % confidence intervals (95 % CI) estimated for each sex separately, and unexponentiated constant terms

**Variable**	**Women (n=7438)**		**Men (n=6053)**	
	**Odds ratio**	**(95% CI)**	**Odds ratio**	**(95% CI)**
Age (years)				
71-73	1.00	ref	1.00	ref
74-76	1.47	(1.20 to 1.79)	1.65	(1.46 to 1.87)
77-79	2.03	(1.64 to 2.51)	2.47	(2.16 to 2.84)
Smoking status				
Never smoked	1.00	ref	1.00	ref
Ex-smoker	1.45	(1.29 to 1.63)	1.54	(1.35 to 1.75)
Current smoker	2.33	(1.85 to 2.93)	2.68	(2.19 to 3.29)
BMI (kg/m^2^)				
Normal(18.5 to <25)	1.00	ref	1.00	ref
Overweight (25 to <30)	0.82	(0.72 to 0.92)	0.87	(0.77 to 0.99)
Obese (≥30)	1.03	(0.88 to 1.21)	0.98	(0.83 to 1.15)
Alcohol consumption				
Weekly or more	1.00	ref	1.00	ref
Less than weekly	1.27	(1.12 to 1.44)	1.23	(1.10 to 1.38)
Physical activity (MET.minutes/week)				
Inactive (<600)	1.00	ref	1.00	ref
Active (≥600)	0.66	(0.58 to 0.74)	0.68	(0.61 to 0.76)
Constant (not exponentiated)	−1.76	(−1.98 to −1.53)	−1.07	(−1.24 to −0.90)
Absolute risk %	14.7	(12.1 to 17.8)	25.6	(22.4 to 29.1)

### Absolute risk of death

**Figure 1:**
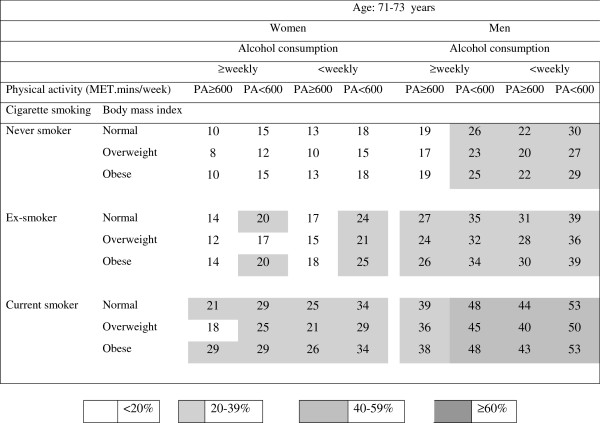
**Absolute risk chart for women and men aged 71–73 years for death within 10 years; the numbers in the cells are the predicted risk (%) calculated from the logistic regression model summarized in Table**2.

**Figure 2:**
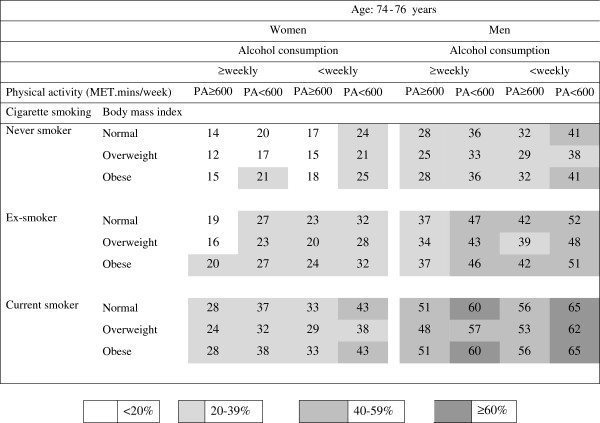
**Absolute risk chart for women and men aged 74–76 years for death within 10 years; the numbers in the cells are the predicted risk (%) calculated from the logistic regression model summarized in Table**2.

**Figure 3:**
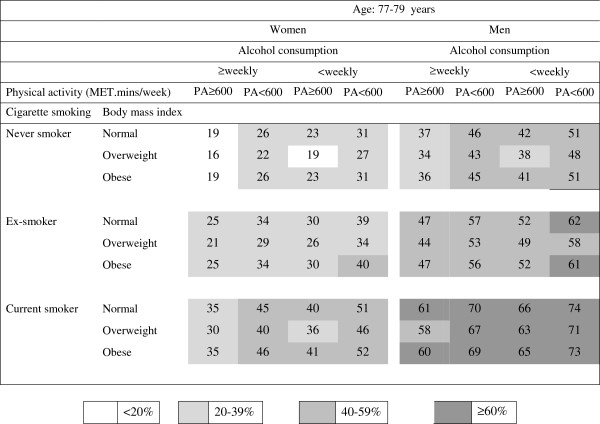
**Absolute risk chart for women and men aged 77–79 years for death within 10 years; the numbers in the cells are the predicted risk (%) calculated from the logistic regression model summarized in Table**2.

The predicted risk estimates (shown as percentages) for 10-year mortality were calculated from the results shown in Table 2 and are presented in the risk charts in Figures 1, 2 and 3.

In each age and sex category, risks were lowest in overweight, physically active, never smokers who drank alcohol at least weekly and risks were highest in physically inactive, current smokers who drank alcohol less than weekly (including never or rarely) and were in either the normal weight or obese categories.

## Discussion

By combining data from the major behavioural risk factors in the context of the very substantial effects of age and sex, the absolute risk charts presented here provide a 'real world' overview of the combined effects of risk factors on mortality in older people.

The strengths of our study are that it includes a large number of men and women drawn from population databases, who are representative of older Australian men and women in the community. Follow-up was complete for all deaths and a large number of outcomes (deaths) were recorded.

There are several potential limitations of the study. First, our samples were confined to older Australians so the results may not be generalizable to other populations, or for men living outside major cities. Second, data were only available for demographic and behavioural factors and not for biomedical markers such as blood pressure, lipid levels, or chronic conditions such as diabetes. Third, there were small differences in wording in the physical activity variable, however it is unlikely that these would have substantively impacted on the calculation of MET.minutes. Fourth, post-survey review of data relating to BMI and smoking resulted in fewer missing data for the men and differences in age ranges between men and women meant that not all data could be used for this analysis, although the overall pattern of estimates is consistent with hazard ratios we have reported previously [7,19]. Finally, as in all observational studies, bias and confounding cannot be eliminated. Reverse causality bias could underpin the association between decreased alcohol intake and increased mortality (i.e., presence of disease could increase risk of death and also lead to decreased alcohol intake) and could attenuate any association between obesity and mortality (i.e., presence of disease could increase risk of death and also lead to weight loss). However both these explanations are unlikely, given the long period of follow-up, and that separate more detailed survival analyses of alcohol use and BMI among these cohorts showed a similarly higher mortality among non-drinkers and those who were obese [7,19].

## Conclusions

Our results extend previous work reporting on the combined health effects of health behaviours for older people [24]. Our absolute risk charts for mortality in elderly Australians, based on combined demographic and behavioural risk factors, provide simple tools for communicating risk. They complement other absolute risk charts published for clinical use, especially for cardiovascular disease [14,15,25,26], which do not cover for all-cause mortality in older adults.

## Competing interests

All authors have completed the Unified Competing Interest form at http://www.icmje.org/coi_disclosure.pdf (available on request from the corresponding author) and all authors want to declare (1) Financial support for the submitted work from the National Health and Medical Research Council and the Australian Government Department of Health and Ageing. All authors also declare (2) No financial relationships with commercial entities that might have an interest in the submitted work; (3) No spouses, partners or children with relationships with commercial entities that might have an interest in the submitted work; (4) No non-financial interests that may be relevant to the submitted work.

## Authors’ contributions

AD, LF and WB devised the idea for the study. AD was responsible for the statistical analysis. All authors were involved in interpreting the results of the analysis and critically reviewed the manuscript. The final version was approved by all authors. AD is the guarantor. All authors read and approved the final manuscript.

### Funding

The Men, Women and Ageing project is funded by a National Health and Medical Research Council of Australia /Australian Research Council Ageing Well, Ageing Productively Strategic Award (409953). The Australian Longitudinal Study on Women’s Health is funded by the Australian Government Department of Health and Ageing. The Health in Men Study is supported by grants from the National Health and Medical Research Council of Australia (project grant numbers 279408, 379600, 403963). The funding sources had no role in the design and conduct of the study; collection, management, analysis, and interpretation of the data; or preparation, review, or approval of the manuscript.

## Pre-publication history

The pre-publication history for this paper can be accessed here:

http://www.biomedcentral.com/1471-2458/12/669/prepub
